# Health Care Access and Use Among Children & Adolescents Exposed to Parental Incarceration—United States, 2019

**DOI:** 10.1016/j.acap.2022.10.001

**Published:** 2022-10-08

**Authors:** Rohan Khazanchi, Nia J. Heard-Garris, Tyler N.A. Winkelman

**Affiliations:** Health, Homelessness, and Criminal Justice Lab, Hennepin Healthcare Research Institute, Minneapolis, Minn; School of Public Health, University of Minnesota, Minneapolis, Minn; College of Medicine, University of Nebraska Medical Center, Omaha, Nebr; Division of Advanced General Pediatrics, Department of Pediatrics and Mary Ann & J. Milburn Smith Child Health Research, Outreach, and Advocacy Center, Stanley Manne Children’s Research Institute, Ann & Robert H. Lurie Children’s Hospital, Chicago, Ill; Department of Pediatrics, Northwestern University Feinberg School of Medicine, Chicago, Ill; Institute for Policy Research, Northwestern University, Chicago, Ill; Health, Homelessness, and Criminal Justice Lab, Hennepin Healthcare Research Institute, Minneapolis, Minn; Division of General Internal Medicine, Department of Medicine, Hennepin Healthcare, Minneapolis, Minn

**Keywords:** access to care, adverse childhood experiences (ACEs), health utilization, mental health, parental incarceration (PI)

## Abstract

**Objective:**

The United States has the highest incarceration rate in the world, with spillover impacts on 5 million children with an incarcerated parent. Children exposed to parental incarceration (PI) have suboptimal health care access, use, and outcomes *in adulthood* compared to their peers. However, little is known about their access and utilization *during childhood.* We evaluated relationships between PI and health care use and access throughout childhood and adolescence.

**Methods:**

We analyzed the nationally representative 2019 National Health Interview Survey Child Sample to examine cross-sectional associations between exposure to incarceration of a residential caregiver, access to care, and health care use among children aged 2–17. Respondents were asked about measures of preventive care access, unmet needs due to cost, and acute care use over the last year. We estimated changes associated with PI exposure using multiple logistic regression models adjusted for age, sex, race, ethnicity, parental education, family structure, rurality, income, insurance status, and disability.

**Results:**

Of 7,877 sample individuals representing a weighted population of 63,046,969 children, 484 (weighted 3,761,207; 6.0% [95% CI 5.4–6.6]) were exposed to PI. In adjusted analyses to produce national estimates, exposure to PI was associated with an additional 123,703 children lacking a usual source of care, 114,795 with forgone dental care needs, 75,434 with delayed mental health care needs, and 53,678 with forgone mental health care needs.

**Conclusions:**

Exposure to PI was associated with worse access to a usual source of care and unmet dental and mental health care needs. Our findings highlight the need for early intervention by demonstrating that these barriers emerge during childhood and adolescence.

Incarceration rates in the US have increased fivefold since 1970.^1^ With over 2.2 million people incarcerated on any given day, the United States maintains the highest incarceration rate (700 per 100,000) in the world.^1^ As a result, over 5 million kids—7% of all US children—have had a parent who lived with them go to jail or prison.^2,3^ Parental incarceration (PI) is disproportionately concentrated among Black, poor, and rural children, as well as among children of parents with low educational attainment.^4,5^ Importantly, the inequitable and racialized distribution of PI can lead to other adverse exposures, including child poverty.^6^

Exposure to PI has been identified as a key adverse childhood experience (ACE) with physical and mental health impacts across the life course.^7,8^ Moreover, children with one or more incarcerated parent are exposed to nearly five times as many other ACEs as their counterparts without incarcerated parents.^9^ This is especially concerning given the additive, dose-response impact of ACEs on health.^7,10^ PI exposure is independently associated with increased incidence of learning and developmental disabilities, physical health conditions, and mental health conditions in adulthood.^8,11,12^ Exposure to PI is also associated with worse access to health care in young adulthood; a longitudinal study using National Longitudinal Study of Adolescent to Adult Health (Add Health) data from 1995 to 2008 found increased odds of forgone medical care among young adults (24–32 years old) exposed to paternal incarceration. In the same study, exposure to maternal incarceration was associated with increased odds of forgone medical care and lacking a usual source of primary care.^5^

However, very little is known about the health care access and use of children and adolescents exposed to PI *during childhood itself*. This may be a missed opportunity for early intervention, given that access to care and unmet health care needs in childhood independently predict adult health behaviors and outcomes.^13,14^ A cross-sectional study of the 2011–2012 National Survey of Children’s Health (NSCH) data found substantial unmet mental health care needs among children exposed to PI.^15^ Yet, given that health care coverage and access for children have improved significantly over the last decade across a number of relevant indicators,^16^ older evaluations with limited measures of access and utilization may not necessarily reflect the current context. This is especially pertinent after implementation of the Affordable Care Act (ACA) in 2010 and Medicaid expansions in 2014, which substantially increased coverage for adults with criminal justice involvement and had broadly documented spillover impacts on key child health and access to care indicators.^17–20^ Thus, the current state of health care access and use during childhood and adolescence among those exposed to PI remains ill-defined.

In this study, we leveraged a new biennial question about PI added to the 2019 National Health Interview Survey (NHIS), a gold standard evaluation of health care access and use.^21^ Among this nationally representative cohort, we assessed the association of PI with health care access and use to determine the scale and scope of barriers among children and adolescents exposed to PI. Our a priori hypothesis, based on existing literature,^5,15^ was that children and adolescents exposed to PI would have worse access to preventive care, higher rates of delayed or forgone care, and increased utilization in acute care settings compared to those who had not experienced PI.

## METHODS

### Study Design and Data Source

We conducted a cross-sectional study of the 2019 NHIS Child Sample to examine the association of PI with measures of health care access and use. Institutional review board approval was not required for this secondary analysis of a publicly available and nonidentifiable dataset. This study follows the Strengthening the Reporting of Observational Studies in Epidemiology (STROBE) reporting guidelines.

### Study Sample

The NHIS Child Sample is a nationally representative, cross-sectional survey of the noninstitutionalized, civilian population across all 50 US states and the District of Columbia.^21^ The NHIS uses geographically clustered sampling techniques to select a sample of dwelling units. Within each sample household, information was obtained from in-person interviews with a parent or adult knowledgeable about and responsible for the health care of one randomly selected child aged 0 to 17 years. The NHIS Child Sample response rate for 2019 was 59.1%. Details about the sampling methodology and the specific phrasing of the 2019 NHIS Child Sample questionnaire are available publicly online.^21^

We excluded children less than 2 years of age from our sample, as measures of health care access and use were not assessed for this subpopulation. Respondents who refused to answer, whose answers were not ascertained, or who did not know the answer to a given question were recorded as “missing” for that item.

### Measures

#### Independent Variable: Parental Incarceration

As part of the rotating core NHIS questions on Stressful Life Events initiated in 2019, adult respondents were asked whether the sample child “ever lived with a parent or guardian who served time in jail or prison after they were born.” This variable captures both current and previous parental incarceration, but only captures incarceration of a residential parent and thus may underestimate overall prevalence of PI.^5,12^ Nevertheless, capturing residential parental incarceration may be especially important since these children experience worse outcomes than children with a nonresidential incarcerated parent.^22^ This question may also capture exposure to incarceration of a nonparental guardian (eg, grandparent).

#### Dependent Variables: Health Care Access and Use

We selected key dependent variables from questions asked for all sample children aged 2–17 across four primary domains of interest: 1) preventive care access (including having a usual source of care, well visit, and routine dental cleaning in the last year), 2) delayed care due to cost (including dental, medical, and mental health care), 3) forgone care due to cost (including dental, medical, and mental health care), and 4) acute care use (hospitalization, urgent care clinic use, or hospital emergency room use in the last year).

#### Covariates

Using the Andersen Health Utilization Model and the Gelberg-Andersen Behavioral Model for Vulnerable Populations as grounding conceptual frameworks, we identified sociodemographic and clinical factors which might confound the relationships between PI exposure and health care access or health service use.^23,24^ These applied conceptual models encompass characteristics which are *predisposing, enabling*, and *need-based* dynamics that lead to health utilization. *Predisposing factors*, in addition to history of PI, included age (early childhood: 2-<6 years, middle childhood: 6–<12 years, early adolescence: 12–<18; based on National Institute of Child Health & Human Development standards), sex assigned at birth (male, female), self-reported race and ethnicity groups (non-Hispanic white, Hispanic, non-Hispanic Black, Other [including non-Hispanic Asian, non-Hispanic American Indian/Alaska Native, and non-Hispanic multirace, combined for regression analyses due to the small number of observations]) given the multilevel impacts of racism on access to care for minoritized groups,^25^ maximum parental education (less than high school, high school diploma or equivalent, post-secondary) and family structure (2-parent household, 1-parent, no residential parents). *Enabling factors* included rurality (metropolitan, non-metropolitan, based on the 2013 National Center for Health Statistics Urban-Rural classification scheme,^26^ included because of higher documented incarceration rates in rural settings^1,4^), household income (poor: <100% of the federal poverty level [FPL], near-poor: 100–199% FPL, not poor: ≥200% FPL, based on prior studies of child health care access using NHIS^16^), and insurance status (private or military; Medicaid, CHIP, or other public; uninsured currently or anytime in the last 12 months). Lastly, the *need-based factor* included was a validated ecobiodevelopmental measure of disability (“Yes,” “No” for the Washington Group Short Set Composite Disability Indicator, which defines presence of disability based both on the person’s individual functional limitations and their experiences with environmental/societal barriers).

### Statistical Analysis

First, we created our study sample of children aged 2–17 years by selecting respondents with non-missing data for all variables of interest. We summarized descriptive statistics for our study population to document the weighted prevalence of parental incarceration and outcomes of interest. We compared the weighted associations of each variable and PI with χ^2^ tests. Then, for each outcome measure of health care access and use, we constructed multiple logistic regression models to estimate associations with exposure to PI. Each regression included adjustment for all predisposing, enabling, and need-based characteristics measured. We used these models to identify the percentage-point (PP) difference in adjusted marginal effects of PI on each outcome of interest, with our results presented as predicted probabilities by PI exposure and predicted number of children experiencing each outcome.^27^

We performed all analyses using Stata, version 17 (Stata-Corp, College Station, TX), accounted for the clustered, stratified complex survey design, and used poststratification survey weights to produce nationally representative estimates for the population of non-institutionalized, housed children aged 2 to 17 years in the United States. We considered two-tailed *P* < .05 to be statistically significant.

## RESULTS

### Descriptive Statistics and Univariate Analyses

Of 8,188 sample children aged 2–17 in the 2019 NHIS Child Sample, we excluded 311 (3.8%) with missing data for key variables. Of the 7,877 remaining individuals representing a total weighted population of 63,046,969 children, 484 (weighted 6.40% [95% CI 5.4–6.6]) had been exposed to incarceration of a residential caregiver representing a weighted subpopulation of 3,761,207 children. In Table 1, we describe the sociodemographic characteristics of our sample by PI exposure. Notably, exposure to PI was significantly associated with adolescent age, non-Hispanic Black race, lower parental educational attainment, zero or one parent in the household, nonmetropolitan residence, poverty or near-poverty, enrollment in Medicaid or other public insurance, and a positive disability screen. Sex and uninsurance were similar between the 2 groups.

In Table 2, we compare the weighted prevalence of each access and utilization outcome between children exposed and not exposed to PI. In these bivariate analyses, those exposed to PI were more likely to lack a usual place of care but were more likely to have had a routine dental cleaning within the last year (*P* < .05). There was no significant difference in the likelihood of having a well visit within the last year. Delayed and forgone medical, mental health, and dental care due to cost were all more common among those exposure to PI (*P* < .05). Lastly, emergency department use and overnight hospitalization were more common among those exposed to PI (*P* < .05), but there was no significant difference in urgent care use.

### Access to Care, Health Care Use, and Parental Incarceration

We display our adjusted models in Table 3 with corresponding weighted population differences to highlight the predicted number of children and adolescents whose outcome would have differed without exposure to PI. Exposure to PI was associated with 123,703 children and adolescents lacking a usual source of care (PI: 10.1% vs No PI: 6.8%, adjusted difference 3.3 percentage-points [PP]; [95% confidence interval 0.1,6.5]), 75,434 delaying mental health care due to cost (2.9% vs 0.8%, adjusted difference 2.0 PP [0.5,3.5]), 114,795 forgoing needed dental care due to cost by (6.9% vs 3.8%, adjusted difference 3.1 PP [0.2,5.9]), and 53,678 forgoing needed mental health care due to cost (2.4% vs 1.0%, adjusted difference 1.4 PP [0.1,2.7]). In adjusted analyses, there were not statistically significant differences in the probability of having no well visit or routine dental visit, delaying dental or medical care due to cost, forgoing medical care needs due to cost, and being hospitalized or seen at an emergency department. Supplemental Appendices 1–12 contain bivariate and multiple logistic regression models for each outcome, including the exponentiated coefficients (odds ratios) and 95% CIs for all included covariates.

## DISCUSSION

In this contemporary, nationally representative study of children and adolescents ages 2–17, we performed an indepth analysis of PI and health care access and use by analyzing responses to a novel question about PI on the 2019 NHIS. We found that suboptimal access to care associated with PI exposure begins within childhood itself, corroborating and extending prior work examining care utilization in adulthood.^5^ We noted that children exposed to PI were more likely to use the emergency department or be hospitalized overnight in bivariate analyses, but this association was not robust after accounting for other explanatory factors. In covariate-adjusted analyses, we estimated that exposure to parental incarceration was associated with an additional 123,703 children with no usual source of care, 114,795 with forgone dental care needs, 75,434 with delayed mental health care needs, and 53,678 with forgone mental health care needs. Even after large coverage expansions and striking improvements in children’s health care access over the last two decades,^16,18–20^ access to preventive, mental health, and primary care remains challenging and inaccessible for many children and adolescents exposed to PI. As interest in the downstream implications of ACEs continues to grow, the opportunity to prevent PI exposure and sustainably support children exposed to PI cannot be overlooked.^28^

Prior work has highlighted poor access to a usual source of care^5^; unmet dental care needs^15,29^; poor oral health^29^; unmet mental health care needs^5,15^; and increased incidence of depression, anxiety, post-traumatic stress disorder, substance misuse, and suicidality in young adulthood among individuals exposed to childhood PI.^5,11,12^ However, existing literature has relied upon outdated nationally representative data sources, limited health care access and use outcomes, limited adjustment for key confounding factors, and examination of downstream impacts on access to care and utilization during adulthood rather than impacts during childhood itself.^8^ While studies have long identified that material hardship and insurance status may partially explain the association of PI exposure with health care access and outcomes, our study documents an independent association with PI and extends the literature with several novel secondary findings. First, although children who were uninsured in the last year are more likely to lack a usual source of care and forgo or delay needed care,^30^ we found no significant difference in uninsurance between children exposed or unexposed to PI. Second, although children exposed to PI were more likely to be enrolled in Medicaid, CHIP, or other public insurance, insurance status did not fully mediate associations between PI exposure and worse access to a usual source of care, dental care, and mental health care, even in a sample timeframe which includes the documented spillover benefits of post-ACA Medicaid expansions.^18–20^ Lastly, our findings complement a body of research highlighting unmet mental health care needs and increased prevalence of mental health conditions during adolescence and young adulthood^5,11,12^ by showing that disparate rates of delayed and forgone mental health care begin during childhood itself for individuals exposed to PI.

Overall, our findings support calls for continued evaluation and structural intervention to address care disruption spanning from childhood to early adulthood among individuals exposed to PI. Our findings reflect the most recently updated national estimates and demonstrate a striking persistence of poor access to a usual source of care and substantive unmet care needs, corroborating an overall lack of improvement in targeted support for children exposed to PI across the last several decades.^4^ Suboptimal access to care among children exposed to PI reflects a myriad of intertwined social and structural risk conditions, but our study highlights that differences in age, sex, race/ethnicity, educational attainment, family structure, rurality, poverty, insurance status, and disability do not fully explain worse access to care associated with PI. Thus, interventions to improve the health care access, use, and outcomes of children and adolescents exposed to PI will require multifaceted strategies to target both risk and protective factors across multiple levels.^31^

Screening for ACEs like PI cannot be viewed as a standalone approach.^32^ Policy interventions must complement clinical screening tools to consider the “whole child” in the context of their families, schools, communities, and environments.^28^ Clinicians can leverage this framework to interrupt intergenerational transmission of ACEs by integrating care for families through caregiver mental health screening.^28,33^ Moreover, at institutional and policy levels, families are likely to need multiple fronts of support around periods of parental incarceration. Carceral facilities should train staff in family centered practices and on the impact of PI on children; ensure parental needs are assessed at intake and used for linkage to jail and community resources; support family friendly contact, non-contact, video, and phone visits between parents, their children, and systems that impact their children; implement evidence-based parent management training programs; involve caregivers in facility programming; and include caregivers and children in reentry planning.^34–37^ Policymakers should advocate for community investments which *prevent* ACE exposure as a means of improving health outcomes^38,39^ and support upstream interventions for children exposed to PI to enhance school readiness, address food and economic insecurity, and meet basic unmet social needs.^6,40,41^ In short, we must shift from solely identifying ACEs as an individual-level risk condition to recognizing ACEs as consequences and exacerbators of structural trauma.

Strengths of this study include our use of a novel, nationally representative data source on PI. The prevalence of PI exposure in our 2019 NHIS sample (6.0% for children and adolescents 2–17 years) is comparable to point prevalence estimates from recent analyses of the 2016–2018 NSCH (6.4% for children 0–17 years) and 1994–2008 Add Health (9.1% for adolescents 12–19 years).^31,42^ Thus, the NHIS may be a reliable data source to glean new information about PI exposure and evaluate interventions, especially given plans for biennial repeated measurement. Limitations of the study include, first, the use of parent-reported measures, which may contribute to underreporting because of social desirability bias and stigma, though proxy-report is a generally considered a reliable and valid approach for measuring child health care access and use since guardian perceptions strongly influence health services use.^43^ Second, the measurement of “parental incarceration” as a variable describing one or more parent/guardian must be interpreted reasonably, since other studies have documented possible differential impacts of paternal, maternal, and both-parent incarceration on health and health care use,^5,8,11^ though they were limited by small survey samples of individuals reporting maternal or both-parent incarceration. Third, as the NHIS measure of PI is a binary indicator, we were unable to capture the differential impacts of type (e.g., jail versus prison incarceration), duration, or timing of PI.

## CONCLUSIONS

Exposure to PI is associated with worse access to a usual source of care and unmet dental and mental health care needs during childhood and adolescence, even after controlling for a number of predisposing, enabling, and need-based factors associated with health care utilization including insurance status. Poor access may contribute to poor health outcomes within childhood and across the life course for individuals exposed to PI. Trauma-informed, cross-sector care delivery innovations are needed to incentivize partnership between jails, prisons, policymakers, and clinicians which mitigate these immediate and life-course implications. Moreover, policymakers should consider how upstream interventions to ameliorate persistently high rates of incarceration in the United States could reduce childhood PI exposure, diminish downstream costs, and prevent adverse health consequences.

## Supplementary Material

mmc1

## Figures and Tables

**Table 1.** Weighted Descriptive Statistics for Characteristics of Respondents Exposed and Not Exposed to Parental Incarceration (PI)

	Exposed to PI		Not Exposed to PI		χ2 Test
	Weighted N = 3,761,207 (484 Observations)		Weighted N = 59,285,761 (7,393 Observations)		
	Proportion	95% CI	Proportion	95% CI	*P*-value
**Age Category**					
Early Childhood (2 - <6 y)	16.4	[12.4,21.3]	25.2	[23.9,26.4]	**0.003**
Middle Childhood (6 - <12 y)	39.7	[34.7,45.0]	36.9	[35.5,38.3]	
Adolescence (12 - <18 y)	43.9	[38.5,49.4]	38.0	[36.6,39.4]	
**Sex**					
Female	49.9	[44.3,55.5]	49.2	[47.8,50.5]	0.814
Male	50.1	[44.5,55.7]	50.8	[49.5,52.2]	
**Race/Ethnicity**					
NH White	51.4	[45.6,57.2]	51.9	[49.9,54.0]	0.214
Hispanic	22.2	[17.8,27.2]	25.8	[23.8,27.9]	
NH Black	16.0	[12.3,20.6]	12.4	[11.2,13.8]	
Other (NH Asian, NH AIAN, Other/Multiracial)	10.4	[7.1,14.9]	9.8	[8.8,10.9]	
**Highest Level of Parental Educational Attainment**					
Less than high school	11.1	[8.2,14.9]	8.3	[7.2,9.4]	**<0.001**
High school, GED, or equivalent	25.2	[20.6,30.5]	18.8	[17.6,20.1]	
Post-secondary education	52.2	[46.8,57.5]	71.9	[70.2,73.5]	
**Number of Parents in Household**					
No parents in household	11.5	[8.4,15.5]	1.1	[0.8,1.3]	**<0.001**
1 parent	57.3	[51.7,62.7]	28.4	[27.0,29.9]	
2+ parents	31.3	[26.2,36.9]	70.5	[69.0,71.9]	
**Rurality** ^*^					
Nonmetropolitan	21.4	[17.1,26.6]	13.2	[11.8,14.8]	**<0.001**
Metropolitan	78.6	[73.4,82.9]	86.8	[85.2,88.2]	
**Family Income as % of Federal Poverty Level**					
Poor (<100% FPL)	31.7	[26.7,37.1]	16.1	[14.8,17.4]	**<0.001**
Near-Poor (100% to 199% FPL)	31.9	[26.8,37.5]	22.7	[21.4,24.0]	
Not Poor (>=200% FPL)	36.4	[31.3,41.9]	61.2	[59.4,63.1]	
**Primary Source of Health Insurance**					
Private or military	22.8	[18.8,27.4]	60.4	[58.6,62.3]	**<0.001**
Medicaid, CHIP, or other public	67.6	[62.5,72.3]	31.9	[30.2,33.6]	
Uninsured	9.6	[6.8,13.4]	7.7	[6.9,8.6]	
**Washington Group Short Set Composite Disability Indicator**					
No	78.1	[73.5,82.1]	90.3	[89.4,91.2]	**<0.001**
Yes	21.9	[17.9,26.5]	9.7	[8.8,10.6]	

Proportion = weighted column percent; 95% CI = 95% Confidence Interval, based on Standard Errors computed using Taylor Series; NH = non-Hispanic; AIAN = American Indian or Alaskan Native.

*P*-values in **bold** reflect statistical significance at *P*<0.05.

* Rurality is based on the 2013 NCHS Urban-Rural Classification Scheme for Counties.

**Table 2.** Weighted Descriptive Statistics for Access to Care and Health Care Use among Respondents Exposed and Not Exposed to Parental Incarceration (PI)

	Exposed to PI		Not exposed to PI		χ2 Test
	Weighted N = 3,761,207 (484 Observations)		Weighted N = 59,285,761 (7,393 Observations)		
	Proportion	95% CI	Proportion	95% CI	*P*-value
**Usual place of care**					
Has a usual place of care (doctor’s office)	87.7	[83.8,90.8]	93.2	[92.5,94.0]	**<0.001**
Does not have a usual place of care (urgent care, emergency	12.3	[9.2,16.2]	6.8	[6.0,7.5]	
room, other, or no usual place of care)					
**Well visit, past 12m**					
Had a well visit	92.6	[89.5,94.8]	93.2	[92.4,93.8]	0.661
No well visit	7.4	[5.2,10.5]	6.8	[6.2,7.6]	
**Routine dental cleaning, past 12m**					
Had routine dental cleaning	96.8	[94.3,98.3]	93.1	[92.3,93.9]	**0.009**
No dental cleaning	3.2	[1.7,5.7]	6.9	[6.1,7.7]	
**Delayed dental care d/t cost, past 12m**					
Did not delay dental care	91.4	[87.6,94.1]	94.7	[94.0,95.4]	**0.016**
Delayed dental care	8.6	[5.9,12.4]	5.3	[4.6,6.0]	
**Delayed medical care d/t cost, past 12m**					
Did not delay medical care	96.8	[93.9,98.3]	98.7	[98.4,99.0]	**0.011**
Delayed medical care	3.2	[1.7,6.1]	1.3	[1.0,1.6]	
**Delayed mental health care d/t cost, past 12m**					
Did not delay mental health care	95.6	[92.9,97.4]	99.2	[98.9,99.4]	**<0.001**
Delayed mental health care	4.4	[2.6,7.1]	0.8	[0.6,1.1]	
**Needed dental care but did not get it d/t cost, past 12m**					
No forgone dental care needs	91.4	[87.7,94.1]	96.2	[95.6,96.7]	**<0.001**
Forgone dental care needs	8.6	[5.9,12.3]	3.8	[3.3,4.4]	
**Needed medical care but did not get it d/t cost, past 12m**					
No unmet medical care needs	96.7	[93.9,98.3]	99	[98.7,99.2]	**0.001**
Forgone medical care needs	3.3	[1.7,6.1]	1	[0.8,1.3]	
**Needed mental health care but did not get it d/t cost, past 12m**					
No unmet mental health care needs	96.2	[93.6,97.7]	99.1	[98.8,99.3]	**<0.001**
Forgone mental health care needs	3.8	[2.3,6.4]	0.9	[0.7,1.2]	
**Visited urgent care, past 12m**					
0 visits	73.3	[68.4,77.6]	72.7	[71.2,74.2]	0.815
1+ visit(s)	26.7	[22.4,31.6]	27.3	[25.8,28.8]	
**Visited hospital ED, past 12m**					
0 visits	77.6	[72.9,81.7]	82.5	[81.4,83.7]	**0.019**
1+ visit(s)	22.4	[18.3,27.1]	17.5	[16.3,18.6]	
**Hospitalized overnight, past 12m**					
Never hospitalized	95.2	[92.5,97.0]	97.6	[97.2,98.0]	**0.006**
Hospitalized	4.8	[3.0,7.5]	2.4	[2.0,2.8]	

Proportion = weighted column percent; 95% CI = 95% Confidence Interval, based on Standard Errors computed using Taylor Series.

*P*-values in **bold** reflect statistical significance at *P*<0.05.

**Table 3.** Association of Exposure to Parental Incarceration with Access to Care and Health Care Use

	Exposed to PI		Not exposed to PI		Adjusted Difference^†^			
	Predictive margins*	95% CI	Predictive margins*	95% CI	Percentage-point difference	95% CI	Weighted population difference^‡^	*P*-value^§^
**Preventive Care Access**								
No usual source of care	10.1	[7.0,13.3]	6.8	[6.1,7.6]	3.3	[0.1,6.5]	123,703	**0.044**
No well visit	7.2	[4.7,9.7]	6.9	[6.1,7.6]	0.3	[−2.2,2.8]	12,302	0.796
No dental visit	5.0	[2.1,7.9]	6.7	[6.0,7.5]	−1.8	[−4.7,1.2]	−65,903	0.250
**Delayed Care due to Cost**								
Delayed dental care	8.1	[5.0,11.2]	5.3	[4.6,6.0]	2.8	[−0.3,6.0]	105,010	0.083
Delayed medical care	2.3	[0.7,4.0]	1.3	[1.0,1.6]	1.0	[−0.7,2.7]	36,926	0.251
Delayed mental health care	2.9	[1.4,4.3]	0.8	[0.6,1.1]	2.0	[0.5,3.5]	75,434	**0.010**
**Forgone Care due to Cost**								
Forgone dental care needs	6.9	[4.1,9.6]	3.8	[3.3,4.4]	3.1	[0.2,5.9]	114,795	**0.035**
Forgone medical care needs	2.2	[0.5,3.8]	1.0	[0.8,1.3]	1.1	[−0.5,2.8]	43,223	0.183
Forgone mental health care needs	2.4	[1.1,3.7]	1.0	[0.7,1.2]	1.4	[0.1,2.7]	53,678	**0.032**
**Acute Care Use**								
Visited urgent care	25.9	[21.1,30.8]	27.3	[25.8,28.9]	−1.4	[−6.5,3.7]	−52,284	0.593
Visited hospital ED	18.4	[14.4,22.4]	17.7	[16.6,18.9]	0.7	[−3.3,4.7]	25,804	0.737
Hospitalized overnight	3.3	[1.7,4.8]	2.4	[2.0,2.9]	0.8	[−0.1,2.5]	31,398	0.325

95% CI = 95% Confidence Interval, based on unconditional Standard Errors. Total Weighted N = 63,046,969 (7,877 Observations).

* Predictive margins and 95% CIs reflect the predicted percent probability of each outcome for individuals with or without exposure to PI. All predictive margins were obtained from logistic regression models adjusted for age category, sex, race/ethnicity, max parental education, family structure, rurality, poverty status, insurance status, and the Washington Group Short Set Composite Disability Indicator. Outputs for bivariate and multiple logistic regression models are displayed in Supplemental Appendices 1–12.

† Adjusted Difference reflects the difference in predictive margins.

‡ Weighted population difference reflects the percentage-point difference multiplied by the weighted size of the population exposed to PI.

§ *P*-values reflect a significance test for the null hypothesis that there is no difference between the predictive margins from the “PI” and “No PI” groups. *P*-values in **bold** reflect statistical significance at *P*<0.05.

## What’s New

Exposure to parental incarceration was associated with worse access to a usual source of care and unmet dental and mental health care needs. This nationally representative study extends existing literature on suboptimal access to care among young adults previously exposed to PI by demonstrating these trends start within childhood.
