# Supplementary material for: Health Care Access and Use Among Children & Adolescents Exposed to Parental Incarceration—United States, 2019
Source: Acad Pediatr. Author manuscript; Available in PMC 2024 Sep 5. (PMC11376232; doi:10.1016/j.acap.2022.10.001)
Supplement: mmc1 [file NIHMS1999989-supplement-mmc1.docx]

**SUPPLEMENTAL APPENDICES**

Health Care Access and Use Among Children & Adolescents
Exposed to Parental Incarceration — United States, 2019

*Rohan Khazanchi, Nia Heard-Garris, Tyler N.A. Winkelman*

PAGE 3  **eTable 1.** Bivariate and Multivariate Logistic Regression Models for the Association of Parental Incarceration Exposure and Lack of Access to a Usual Place of Care

PAGE 4  **eTable 2.** Bivariate and Multivariate Logistic Regression Models for the Association of Parental Incarceration Exposure and Lacking a Well Visit in the Past 12 Months

PAGE 5 **eTable 3.** Bivariate and Multivariate Logistic Regression Models for the Association of Parental Incarceration Exposure and Lacking a Routine Dental Cleaning in the Past 12 Months

PAGE 6 **eTable 4:** Bivariate and Multivariate Logistic Regression Models for the Association of Parental Incarceration Exposure and Delaying Dental Care due to Cost in the Past 12 Months

PAGE 7 **eTable 5:** Bivariate and Multivariate Logistic Regression Models for the Association of Parental Incarceration Exposure and Delaying Medical Care due to Cost in the Past 12 Months

PAGE 8 **eTable 6:** Bivariate and Multivariate Logistic Regression Models for the Association of Parental Incarceration Exposure and Delaying Mental Health Care due to Cost in the Past 12 Months

PAGE 9 **eTable 7:** Bivariate and Multivariate Logistic Regression Models for the Association of Parental Incarceration Exposure and Forgoing Needed Dental Care due to Cost in the Past 12 Months

PAGE 10 **eTable 8:** Bivariate and Multivariate Logistic Regression Models for the Association of Parental Incarceration Exposure and Forgoing Needed Medical Care due to Cost in the Past 12 Months

PAGE 11 **eTable 9:** Bivariate and Multivariate Logistic Regression Models for the Association of Parental Incarceration Exposure and Forgoing Needed Mental Health Care due to Cost in the Past 12 Months

PAGE 12 **eTable 10:** Bivariate and Multivariate Logistic Regression Models for the Association of Parental Incarceration Exposure and Urgent Care Use in the Past 12 Months

PAGE 13 **eTable 11:** Bivariate and Multivariate Logistic Regression Models for the Association of Parental Incarceration Exposure and Emergency Department Use in the Past 12 Months

PAGE 14 **eTable 12:** Bivariate and Multivariate Logistic Regression Models for the Association of Parental Incarceration Exposure and Overnight Hospitalization in the Past 12 Months

**eTable 1.** Bivariate and Multivariate Logistic Regression Models for the Association of Parental Incarceration Exposure and Lacking a Usual Place of Care

| Usual place of care | *Weighted N=58,954,049 (7,383 Observations)* | | | |
| --- | --- | --- | --- | --- |
|  | **Bivariate Model** | | **Multivariate Model** | |
|  | **OR** | **95% CI** | **OR** | **95% CI** |
| **Parental incarceration (ref. = No history of PI)** |  |  |  |  |
| History of PI | 1.931*** | [1.37,2.72] | 1.562* | [1.07,2.27] |
| **Age category (ref. = Early Childhood (2 - <6 y))** |  |  |  |  |
| Middle Childhood (6 - <12 y) |  |  | 1.22 | [0.90,1.66] |
| Adolescence (12 - <18 y) |  |  | 1.468** | [1.11,1.95] |
| **Sex (ref. = Male)** |  |  |  |  |
| Female |  |  | 1.002 | [0.80,1.25] |
| **Race/ethnicity (ref. = NH White)** |  |  |  |  |
| Hispanic |  |  | 1.004 | [0.76,1.33] |
| NH Black |  |  | 0.841 | [0.57,1.25] |
| Other (NH Asian, NH AIAN, Other/Multiracial) |  |  | 1.183 | [0.80,1.74] |
| **Number of parents in household (ref. = 2+ parents)** |  |  |  |  |
| No parents in household |  |  | 1.704 | [0.86,3.37] |
| 1 parent |  |  | 1.038 | [0.80,1.35] |
| **Highest level of parent educational attainment (ref. = Post-secondary education)** |  |  |  |  |
| Less than high school |  |  | 1.990*** | [1.38,2.88] |
| High school, GED, or equivalent |  |  | 1.437* | [1.09,1.90] |
| **Urbanicity (ref. = Metropolitan)** |  |  |  |  |
| Nonmetropolitan |  |  | 0.902 | [0.65,1.26] |
| **Family income (ref. = Non-Poor [>=200% FPL])** |  |  |  |  |
| Poor (<100% FPL) |  |  | 1.321 | [0.90,1.94] |
| Near-Poor (100% to 199% FPL) |  |  | 1.468* | [1.09,1.98] |
| **Primary source of health insurance (ref. = Private or military)** |  |  |  |  |
| Medicaid, CHIP, or other public |  |  | 1.089 | [0.79,1.51] |
| Uninsured |  |  | 3.594*** | [2.61,4.95] |
| **Washington Group Short Set Composite Disability Indicator (ref. = No)** |  |  |  |  |
| Yes |  |  | 0.943 | [0.70,1.27] |
| **Intercept** | 0.072*** | [0.06,0.08] | 0.035*** | [0.03,0.05] |
| * p<0.05, ** p<0.01, *** p<0.001 |  |  |  |  |

**eTable 2.** Bivariate and Multivariate Logistic Regression Models for the Association of Parental Incarceration Exposure and Lacking a Well Visit in the Past 12 Months

| Well visit, past 12m | *Weighted N=58,954,049 (7,383 Observations)* | | | |
| --- | --- | --- | --- | --- |
|  | **Bivariate Model** | | **Multivariate Model** | |
|  | **OR** | **95% CI** | **OR** | **95% CI** |
| **Parental incarceration (ref. = No history of PI)** |  |  |  |  |
| History of PI | 1.088 | [0.74,1.59] | 1.054 | [0.71,1.56] |
| **Age category (ref. = Early Childhood (2 - <6 y))** |  |  |  |  |
| Middle Childhood (6 - <12 y) |  |  | 1.916*** | [1.40,2.63] |
| Adolescence (12 - <18 y) |  |  | 2.360*** | [1.76,3.17] |
| **Sex (ref. = Male)** |  |  |  |  |
| Female |  |  | 0.925 | [0.75,1.14] |
| **Race/ethnicity (ref. = NH White)** |  |  |  |  |
| Hispanic |  |  | 0.877 | [0.65,1.19] |
| NH Black |  |  | 0.805 | [0.53,1.22] |
| Other (NH Asian, NH AIAN, Other/Multiracial) |  |  | 1.197 | [0.87,1.66] |
| **Number of parents in household (ref. = 2+ parents)** |  |  |  |  |
| No parents in household |  |  | 0.669 | [0.29,1.52] |
| 1 parent |  |  | 0.998 | [0.78,1.27] |
| **Highest level of parent educational attainment (ref. = Post-secondary education)** |  |  |  |  |
| Less than high school |  |  | 2.459*** | [1.58,3.82] |
| High school, GED, or equivalent |  |  | 1.177 | [0.86,1.62] |
| **Urbanicity (ref. = Metropolitan)** |  |  |  |  |
| Nonmetropolitan |  |  | 1.395* | [1.03,1.89] |
| **Family income (ref. = Non-Poor [>=200% FPL])** |  |  |  |  |
| Poor (<100% FPL) |  |  | 0.655* | [0.43,0.99] |
| Near-Poor (100% to 199% FPL) |  |  | 0.843 | [0.64,1.11] |
| **Primary source of health insurance (ref. = Private or military)** |  |  |  |  |
| Medicaid, CHIP, or other public |  |  | 1.007 | [0.73,1.39] |
| Uninsured |  |  | 4.509*** | [3.40,5.98] |
| **Washington Group Short Set Composite Disability Indicator (ref. = No)** |  |  |  |  |
| Yes |  |  | 0.778 | [0.55,1.11] |
| **Intercept** | 0.073*** | [0.07,0.08] | 0.033*** | [0.02,0.05] |
| * p<0.05, ** p<0.01, *** p<0.001 |  |  |  |  |

**eTable 3.** Bivariate and Multivariate Logistic Regression Models for the Association of Parental Incarceration Exposure and Lacking a Routine Dental Cleaning in the Past 12 Months

| Routine dental cleaning, past 12m | *Weighted N=58,954,049 (7,383 Observations)* | | | |
| --- | --- | --- | --- | --- |
|  | **Bivariate Model** | | **Multivariate Model** | |
|  | **OR** | **95% CI** | **OR** | **95% CI** |
| **Parental incarceration (ref. = No history of PI)** |  |  |  |  |
| History of PI | 0.444* | [0.24,0.83] | 0.68 | [0.32,1.42] |
| **Age category (ref. = Early Childhood (2 - <6 y))** |  |  |  |  |
| Middle Childhood (6 - <12 y) |  |  | 0.042*** | [0.03,0.06] |
| Adolescence (12 - <18 y) |  |  | 0.028*** | [0.02,0.05] |
| **Sex (ref. = Male)** |  |  |  |  |
| Female |  |  | 0.949 | [0.73,1.23] |
| **Race/ethnicity (ref. = NH White)** |  |  |  |  |
| Hispanic |  |  | 0.765 | [0.52,1.13] |
| NH Black |  |  | 0.925 | [0.59,1.46] |
| Other (NH Asian, NH AIAN, Other/Multiracial) |  |  | 1.106 | [0.75,1.64] |
| **Number of parents in household (ref. = 2+ parents)** |  |  |  |  |
| No parents in household |  |  | 0.208 | [0.04,1.00] |
| 1 parent |  |  | 0.685* | [0.49,0.96] |
| **Highest level of parent educational attainment (ref. = Post-secondary education)** |  |  |  |  |
| Less than high school |  |  | 1.629 | [0.85,3.11] |
| High school, GED, or equivalent |  |  | 0.971 | [0.65,1.46] |
| **Urbanicity (ref. = Metropolitan)** |  |  |  |  |
| Nonmetropolitan |  |  | 1.585* | [1.11,2.25] |
| **Family income (ref. = Non-Poor [>=200% FPL])** |  |  |  |  |
| Poor (<100% FPL) |  |  | 1.589 | [0.99,2.55] |
| Near-Poor (100% to 199% FPL) |  |  | 1.723** | [1.17,2.54] |
| **Primary source of health insurance (ref. = Private or military)** |  |  |  |  |
| Medicaid, CHIP, or other public |  |  | 0.627* | [0.43,0.92] |
| Uninsured |  |  | 2.933*** | [1.90,4.52] |
| **Washington Group Short Set Composite Disability Indicator (ref. = No)** |  |  |  |  |
| Yes |  |  | 0.854 | [0.52,1.41] |
| **Intercept** | 0.074*** | [0.07,0.08] | 0.292*** | [0.23,0.37] |
| * p<0.05, ** p<0.01, *** p<0.001 |  |  |  |  |

**eTable 4:** Bivariate and Multivariate Logistic Regression Models for the Association of Parental Incarceration Exposure and Delaying Dental Care due to Cost in the Past 12 Months

| Delayed dental care d/t cost, past 12m | *Weighted N=58,954,049 (7,383 Observations)* | | | |
| --- | --- | --- | --- | --- |
|  | **Bivariate Model** | | **Multivariate Model** | |
|  | **OR** | **95% CI** | **OR** | **95% CI** |
| **Parental incarceration (ref. = No history of PI)** |  |  |  |  |
| History of PI | 1.691* | [1.10,2.61] | 1.657* | [1.02,2.70] |
| **Age category (ref. = Early Childhood (2 - <6 y))** |  |  |  |  |
| Middle Childhood (6 - <12 y) |  |  | 1.264 | [0.86,1.87] |
| Adolescence (12 - <18 y) |  |  | 1.544* | [1.07,2.23] |
| **Sex (ref. = Male)** |  |  |  |  |
| Female |  |  | 0.997 | [0.78,1.27] |
| **Race/ethnicity (ref. = NH White)** |  |  |  |  |
| Hispanic |  |  | 1.609** | [1.16,2.23] |
| NH Black |  |  | 0.703 | [0.43,1.15] |
| Other (NH Asian, NH AIAN, Other/Multiracial) |  |  | 0.95 | [0.60,1.51] |
| **Number of parents in household (ref. = 2+ parents)** |  |  |  |  |
| No parents in household |  |  | 0.358* | [0.14,0.90] |
| 1 parent |  |  | 1.16 | [0.89,1.52] |
| **Highest level of parent educational attainment (ref. = Post-secondary education)** |  |  |  |  |
| Less than high school |  |  | 0.867 | [0.54,1.38] |
| High school, GED, or equivalent |  |  | 1.066 | [0.75,1.52] |
| **Urbanicity (ref. = Metropolitan)** |  |  |  |  |
| Nonmetropolitan |  |  | 0.664* | [0.45,0.99] |
| **Family income (ref. = Non-Poor [>=200% FPL])** |  |  |  |  |
| Poor (<100% FPL) |  |  | 1.735* | [1.12,2.68] |
| Near-Poor (100% to 199% FPL) |  |  | 1.550** | [1.12,2.14] |
| **Primary source of health insurance (ref. = Private or military)** |  |  |  |  |
| Medicaid, CHIP, or other public |  |  | 0.589** | [0.40,0.87] |
| Uninsured |  |  | 7.232*** | [5.28,9.91] |
| **Washington Group Short Set Composite Disability Indicator (ref. = No)** |  |  |  |  |
| Yes |  |  | 1.682** | [1.23,2.30] |
| **Intercept** | 0.056*** | [0.05,0.06] | 0.024*** | [0.02,0.04] |
| * p<0.05, ** p<0.01, *** p<0.001 |  |  |  |  |

**eTable 5:** Bivariate and Multivariate Logistic Regression Models for the Association of Parental Incarceration Exposure and Delaying Medical Care due to Cost in the Past 12 Months

| Delayed medical care d/t cost, past 12m | *Weighted N=58,954,049 (7,383 Observations)* | | | |
| --- | --- | --- | --- | --- |
|  | **Bivariate Model** | | **Multivariate Model** | |
|  | **OR** | **95% CI** | **OR** | **95% CI** |
| **Parental incarceration (ref. = No history of PI)** |  |  |  |  |
| History of PI | 2.502* | [1.21,5.17] | 1.887 | [0.78,4.58] |
| **Age category (ref. = Early Childhood (2 - <6 y))** |  |  |  |  |
| Middle Childhood (6 - <12 y) |  |  | 1.104 | [0.56,2.17] |
| Adolescence (12 - <18 y) |  |  | 1.54 | [0.79,2.99] |
| **Sex (ref. = Male)** |  |  |  |  |
| Female |  |  | 1.373 | [0.85,2.21] |
| **Race/ethnicity (ref. = NH White)** |  |  |  |  |
| Hispanic |  |  | 1.499 | [0.86,2.61] |
| NH Black |  |  | 1.075 | [0.41,2.84] |
| Other (NH Asian, NH AIAN, Other/Multiracial) |  |  | 1.65 | [0.70,3.87] |
| **Number of parents in household (ref. = 2+ parents)** |  |  |  |  |
| No parents in household |  |  | 0.205 | [0.02,1.73] |
| 1 parent |  |  | 1.813* | [1.08,3.04] |
| **Highest level of parent educational attainment (ref. = Post-secondary education)** |  |  |  |  |
| Less than high school |  |  | 0.677 | [0.30,1.50] |
| High school, GED, or equivalent |  |  | 0.866 | [0.50,1.51] |
| **Urbanicity (ref. = Metropolitan)** |  |  |  |  |
| Nonmetropolitan |  |  | 0.591 | [0.28,1.25] |
| **Family income (ref. = Non-Poor [>=200% FPL])** |  |  |  |  |
| Poor (<100% FPL) |  |  | 1.589 | [0.73,3.44] |
| Near-Poor (100% to 199% FPL) |  |  | 1.222 | [0.67,2.24] |
| **Primary source of health insurance (ref. = Private or military)** |  |  |  |  |
| Medicaid, CHIP, or other public |  |  | 0.74 | [0.33,1.67] |
| Uninsured |  |  | 24.247*** | [13.56,43.35] |
| **Washington Group Short Set Composite Disability Indicator (ref. = No)** |  |  |  |  |
| Yes |  |  | 2.412** | [1.37,4.26] |
| **Intercept** | 0.013*** | [0.01,0.02] | 0.002*** | [0.00,0.00] |
| * p<0.05, ** p<0.01, *** p<0.001 |  |  |  |  |

**eTable 6:** Bivariate and Multivariate Logistic Regression Models for the Association of Parental Incarceration Exposure and Delaying Mental Health Care due to Cost in the Past 12 Months

| Delayed mental health care d/t cost, past 12m | *Weighted N=58,954,049 (7,383 Observations)* | | | |
| --- | --- | --- | --- | --- |
|  | **Bivariate Model** | | **Multivariate Model** | |
|  | **OR** | **95% CI** | **OR** | **95% CI** |
| **Parental incarceration (ref. = No history of PI)** |  |  |  |  |
| History of PI | 5.535*** | [3.07,9.97] | 3.588*** | [1.92,6.72] |
| **Age category (ref. = Early Childhood (2 - <6 y))** |  |  |  |  |
| Middle Childhood (6 - <12 y) |  |  | 3.324 | [0.95,11.66] |
| Adolescence (12 - <18 y) |  |  | 4.021* | [1.16,13.92] |
| **Sex (ref. = Male)** |  |  |  |  |
| Female |  |  | 1.076 | [0.65,1.77] |
| **Race/ethnicity (ref. = NH White)** |  |  |  |  |
| Hispanic |  |  | 0.561 | [0.29,1.10] |
| NH Black |  |  | 0.671 | [0.30,1.50] |
| Other (NH Asian, NH AIAN, Other/Multiracial) |  |  | 0.819 | [0.37,1.83] |
| **Number of parents in household (ref. = 2+ parents)** |  |  |  |  |
| No parents in household |  |  | 1.225 | [0.26,5.80] |
| 1 parent |  |  | 2.338*** | [1.45,3.77] |
| **Highest level of parent educational attainment (ref. = Post-secondary education)** |  |  |  |  |
| Less than high school |  |  | 0.471 | [0.16,1.35] |
| High school, GED, or equivalent |  |  | 1.073 | [0.55,2.09] |
| **Urbanicity (ref. = Metropolitan)** |  |  |  |  |
| Nonmetropolitan |  |  | 0.586 | [0.26,1.33] |
| **Family income (ref. = Non-Poor [>=200% FPL])** |  |  |  |  |
| Poor (<100% FPL) |  |  | 1.402 | [0.62,3.16] |
| Near-Poor (100% to 199% FPL) |  |  | 1.136 | [0.64,2.00] |
| **Primary source of health insurance (ref. = Private or military)** |  |  |  |  |
| Medicaid, CHIP, or other public |  |  | 0.499 | [0.23,1.09] |
| Uninsured |  |  | 2.096* | [1.14,3.85] |
| **Washington Group Short Set Composite Disability Indicator (ref. = No)** |  |  |  |  |
| Yes |  |  | 5.523*** | [3.26,9.35] |
| **Intercept** | 0.008*** | [0.01,0.01] | 0.002*** | [0.00,0.01] |
| * p<0.05, ** p<0.01, *** p<0.001 |  |  |  |  |

**eTable 7:** Bivariate and Multivariate Logistic Regression Models for the Association of Parental Incarceration Exposure and Forgoing Needed Dental Care due to Cost in the Past 12 Months

| Needed dental care but did not get it d/t cost, past 12m | *Weighted N=58,954,049 (7,383 Observations)* | | | |
| --- | --- | --- | --- | --- |
|  | **Bivariate Model** | | **Multivariate Model** | |
|  | **OR** | **95% CI** | **OR** | **95% CI** |
| **Parental incarceration (ref. = No history of PI)** |  |  |  |  |
| History of PI | 2.389*** | [1.53,3.72] | 1.961** | [1.18,3.26] |
| **Age category (ref. = Early Childhood (2 - <6 y))** |  |  |  |  |
| Middle Childhood (6 - <12 y) |  |  | 1.946** | [1.20,3.14] |
| Adolescence (12 - <18 y) |  |  | 2.325*** | [1.45,3.72] |
| **Sex (ref. = Male)** |  |  |  |  |
| Female |  |  | 1.168 | [0.87,1.57] |
| **Race/ethnicity (ref. = NH White)** |  |  |  |  |
| Hispanic |  |  | 1.524* | [1.08,2.15] |
| NH Black |  |  | 0.998 | [0.59,1.68] |
| Other (NH Asian, NH AIAN, Other/Multiracial) |  |  | 1.222 | [0.74,2.02] |
| **Number of parents in household (ref. = 2+ parents)** |  |  |  |  |
| No parents in household |  |  | 0.632 | [0.22,1.80] |
| 1 parent |  |  | 1.212 | [0.90,1.63] |
| **Highest level of parent educational attainment (ref. = Post-secondary education)** |  |  |  |  |
| Less than high school |  |  | 0.872 | [0.51,1.50] |
| High school, GED, or equivalent |  |  | 0.997 | [0.66,1.50] |
| **Urbanicity (ref. = Metropolitan)** |  |  |  |  |
| Nonmetropolitan |  |  | 0.687 | [0.44,1.07] |
| **Family income (ref. = Non-Poor [>=200% FPL])** |  |  |  |  |
| Poor (<100% FPL) |  |  | 1.937** | [1.21,3.10] |
| Near-Poor (100% to 199% FPL) |  |  | 1.421 | [0.97,2.09] |
| **Primary source of health insurance (ref. = Private or military)** |  |  |  |  |
| Medicaid, CHIP, or other public |  |  | 0.775 | [0.50,1.21] |
| Uninsured |  |  | 6.844*** | [4.75,9.86] |
| **Washington Group Short Set Composite Disability Indicator (ref. = No)** |  |  |  |  |
| Yes |  |  | 2.161*** | [1.55,3.01] |
| **Intercept** | 0.039*** | [0.03,0.05] | 0.009*** | [0.01,0.02] |
| * p<0.05, ** p<0.01, *** p<0.001 |  |  |  |  |

**eTable 8:** Bivariate and Multivariate Logistic Regression Models for the Association of Parental Incarceration Exposure and Forgoing Needed Medical Care due to Cost in the Past 12 Months

| Needed medical care but did not get it d/t cost, past 12m | *Weighted N=58,954,049 (7,383 Observations)* | | | |
| --- | --- | --- | --- | --- |
|  | **Bivariate Model** | | **Multivariate Model** | |
|  | **OR** | **95% CI** | **OR** | **95% CI** |
| **Parental incarceration (ref. = No history of PI)** |  |  |  |  |
| History of PI | 3.341** | [1.61,6.93] | 2.315 | [0.91,5.91] |
| **Age category (ref. = Early Childhood (2 - <6 y))** |  |  |  |  |
| Middle Childhood (6 - <12 y) |  |  | 1.389 | [0.62,3.10] |
| Adolescence (12 - <18 y) |  |  | 1.451 | [0.66,3.19] |
| **Sex (ref. = Male)** |  |  |  |  |
| Female |  |  | 1.901* | [1.13,3.19] |
| **Race/ethnicity (ref. = NH White)** |  |  |  |  |
| Hispanic |  |  | 1.416 | [0.76,2.65] |
| NH Black |  |  | 0.562 | [0.16,1.93] |
| Other (NH Asian, NH AIAN, Other/Multiracial) |  |  | 0.806 | [0.31,2.08] |
| **Number of parents in household (ref. = 2+ parents)** |  |  |  |  |
| No parents in household |  |  | 0.219 | [0.03,1.84] |
| 1 parent |  |  | 1.554 | [0.87,2.78] |
| **Highest level of parent educational attainment (ref. = Post-secondary education)** |  |  |  |  |
| Less than high school |  |  | 0.589 | [0.25,1.41] |
| High school, GED, or equivalent |  |  | 0.964 | [0.54,1.72] |
| **Urbanicity (ref. = Metropolitan)** |  |  |  |  |
| Nonmetropolitan |  |  | 0.765 | [0.35,1.66] |
| **Family income (ref. = Non-Poor [>=200% FPL])** |  |  |  |  |
| Poor (<100% FPL) |  |  | 1.592 | [0.74,3.41] |
| Near-Poor (100% to 199% FPL) |  |  | 0.995 | [0.53,1.86] |
| **Primary source of health insurance (ref. = Private or military)** |  |  |  |  |
| Medicaid, CHIP, or other public |  |  | 1.217 | [0.54,2.72] |
| Uninsured |  |  | 21.884*** | [11.70,40.94] |
| **Washington Group Short Set Composite Disability Indicator (ref. = No)** |  |  |  |  |
| Yes |  |  | 3.390*** | [1.90,6.05] |
| **Intercept** | 0.010*** | [0.01,0.01] | 0.001*** | [0.00,0.00] |
| * p<0.05, ** p<0.01, *** p<0.001 |  |  |  |  |

**eTable 9:** Bivariate and Multivariate Logistic Regression Models for the Association of Parental Incarceration Exposure and Forgoing Needed Mental Health Care due to Cost in the Past 12 Months

| Needed mental health care but did not get it d/t cost, past 12m | *Weighted N=58,954,049 (7,383 Observations)* | | | |
| --- | --- | --- | --- | --- |
|  | **Bivariate Model** | | **Multivariate Model** | |
|  | **OR** | **95% CI** | **OR** | **95% CI** |
| **Parental incarceration (ref. = No history of PI)** |  |  |  |  |
| History of PI | 4.193*** | [2.29,7.68] | 2.583** | [1.38,4.84] |
| **Age category (ref. = Early Childhood (2 - <6 y))** |  |  |  |  |
| Middle Childhood (6 - <12 y) |  |  | 2.343 | [0.93,5.91] |
| Adolescence (12 - <18 y) |  |  | 2.624* | [1.08,6.38] |
| **Sex (ref. = Male)** |  |  |  |  |
| Female |  |  | 0.719 | [0.44,1.17] |
| **Race/ethnicity (ref. = NH White)** |  |  |  |  |
| Hispanic |  |  | 0.492* | [0.26,0.94] |
| NH Black |  |  | 0.904 | [0.44,1.84] |
| Other (NH Asian, NH AIAN, Other/Multiracial) |  |  | 0.774 | [0.37,1.62] |
| **Number of parents in household (ref. = 2+ parents)** |  |  |  |  |
| No parents in household |  |  | 1.541 | [0.39,6.14] |
| 1 parent |  |  | 2.120** | [1.36,3.31] |
| **Highest level of parent educational attainment (ref. = Post-secondary education)** |  |  |  |  |
| Less than high school |  |  | 0.579 | [0.21,1.61] |
| High school, GED, or equivalent |  |  | 1.465 | [0.79,2.72] |
| **Urbanicity (ref. = Metropolitan)** |  |  |  |  |
| Nonmetropolitan |  |  | 0.57 | [0.28,1.14] |
| **Family income (ref. = Non-Poor [>=200% FPL])** |  |  |  |  |
| Poor (<100% FPL) |  |  | 1.564 | [0.74,3.29] |
| Near-Poor (100% to 199% FPL) |  |  | 1.014 | [0.59,1.73] |
| **Primary source of health insurance (ref. = Private or military)** |  |  |  |  |
| Medicaid, CHIP, or other public |  |  | 0.542 | [0.27,1.09] |
| Uninsured |  |  | 2.143** | [1.21,3.81] |
| **Washington Group Short Set Composite Disability Indicator (ref. = No)** |  |  |  |  |
| Yes |  |  | 5.624*** | [3.35,9.45] |
| **Intercept** | 0.009*** | [0.01,0.01] | 0.003*** | [0.00,0.01] |
| * p<0.05, ** p<0.01, *** p<0.001 |  |  |  |  |

**eTable 10:** Bivariate and Multivariate Logistic Regression Models for the Association of Parental Incarceration Exposure and Urgent Care Use in the Past 12 Months

| Visited urgent care, past 12m | *Weighted N=58,954,049 (7,383 Observations)* | | | |
| --- | --- | --- | --- | --- |
|  | **Bivariate Model** | | **Multivariate Model** | |
|  | **OR** | **95% CI** | **OR** | **95% CI** |
| **Parental incarceration (ref. = No history of PI)** |  |  |  |  |
| History of PI | 0.971 | [0.76,1.24] | 0.93 | [0.71,1.22] |
| **Age category (ref. = Early Childhood (2 - <6 y))** |  |  |  |  |
| Middle Childhood (6 - <12 y) |  |  | 0.912 | [0.78,1.06] |
| Adolescence (12 - <18 y) |  |  | 0.763*** | [0.65,0.89] |
| **Sex (ref. = Male)** |  |  |  |  |
| Female |  |  | 1.004 | [0.89,1.14] |
| **Race/ethnicity (ref. = NH White)** |  |  |  |  |
| Hispanic |  |  | 0.761** | [0.64,0.91] |
| NH Black |  |  | 0.717** | [0.57,0.91] |
| Other (NH Asian, NH AIAN, Other/Multiracial) |  |  | 0.751** | [0.61,0.93] |
| **Number of parents in household (ref. = 2+ parents)** |  |  |  |  |
| No parents in household |  |  | 0.992 | [0.62,1.60] |
| 1 parent |  |  | 1.363*** | [1.19,1.57] |
| **Highest level of parent educational attainment (ref. = Post-secondary education)** |  |  |  |  |
| Less than high school |  |  | 0.632** | [0.46,0.87] |
| High school, GED, or equivalent |  |  | 0.749** | [0.63,0.89] |
| **Urbanicity (ref. = Metropolitan)** |  |  |  |  |
| Nonmetropolitan |  |  | 0.967 | [0.76,1.23] |
| **Family income (ref. = Non-Poor [>=200% FPL])** |  |  |  |  |
| Poor (<100% FPL) |  |  | 0.805 | [0.63,1.03] |
| Near-Poor (100% to 199% FPL) |  |  | 0.822* | [0.68,0.99] |
| **Primary source of health insurance (ref. = Private or military)** |  |  |  |  |
| Medicaid, CHIP, or other public |  |  | 1.073 | [0.90,1.28] |
| Uninsured |  |  | 0.962 | [0.76,1.22] |
| **Washington Group Short Set Composite Disability Indicator (ref. = No)** |  |  |  |  |
| Yes |  |  | 1.358** | [1.12,1.65] |
| **Intercept** | 0.375*** | [0.35,0.41] | 0.503*** | [0.43,0.59] |
| * p<0.05, ** p<0.01, *** p<0.001 |  |  |  |  |

**eTable 11:** Bivariate and Multivariate Logistic Regression Models for the Association of Parental Incarceration Exposure and Emergency Department Use in the Past 12 Months

| Visited hospital ED, past 12m | *Weighted N=58,954,049 (7,383 Observations)* | | | |
| --- | --- | --- | --- | --- |
|  | **Bivariate Model** | | **Multivariate Model** | |
|  | **OR** | **95% CI** | **OR** | **95% CI** |
| **Parental incarceration (ref. = No history of PI)** |  |  |  |  |
| History of PI | 1.364* | [1.05,1.77] | 1.049 | [0.79,1.39] |
| **Age category (ref. = Early Childhood (2 - <6 y))** |  |  |  |  |
| Middle Childhood (6 - <12 y) |  |  | 0.598*** | [0.49,0.73] |
| Adolescence (12 - <18 y) |  |  | 0.603*** | [0.50,0.72] |
| **Sex (ref. = Male)** |  |  |  |  |
| Female |  |  | 1.039 | [0.89,1.21] |
| **Race/ethnicity (ref. = NH White)** |  |  |  |  |
| Hispanic |  |  | 1.06 | [0.87,1.30] |
| NH Black |  |  | 1.086 | [0.85,1.38] |
| Other (NH Asian, NH AIAN, Other/Multiracial) |  |  | 0.922 | [0.72,1.18] |
| **Number of parents in household (ref. = 2+ parents)** |  |  |  |  |
| No parents in household |  |  | 0.655 | [0.38,1.13] |
| 1 parent |  |  | 1.223* | [1.03,1.46] |
| **Highest level of parent educational attainment (ref. = Post-secondary education)** |  |  |  |  |
| Less than high school |  |  | 0.776 | [0.57,1.05] |
| High school, GED, or equivalent |  |  | 0.854 | [0.70,1.05] |
| **Urbanicity (ref. = Metropolitan)** |  |  |  |  |
| Nonmetropolitan |  |  | 1.078 | [0.86,1.35] |
| **Family income (ref. = Non-Poor [>=200% FPL])** |  |  |  |  |
| Poor (<100% FPL) |  |  | 1.478** | [1.15,1.90] |
| Near-Poor (100% to 199% FPL) |  |  | 1.486*** | [1.20,1.85] |
| **Primary source of health insurance (ref. = Private or military)** |  |  |  |  |
| Medicaid, CHIP, or other public |  |  | 1.484*** | [1.21,1.82] |
| Uninsured |  |  | 1.267 | [0.95,1.68] |
| **Washington Group Short Set Composite Disability Indicator (ref. = No)** |  |  |  |  |
| Yes |  |  | 1.829*** | [1.49,2.25] |
| **Intercept** | 0.212*** | [0.20,0.23] | 0.193*** | [0.16,0.23] |
| * p<0.05, ** p<0.01, *** p<0.001 |  |  |  |  |

**eTable 12:** Bivariate and Multivariate Logistic Regression Models for the Association of Parental Incarceration Exposure and Overnight Hospitalization in the Past 12 Months

| Hospitalization, last 12m | *Weighted N=58,954,049 (7,383 Observations)* | | | |
| --- | --- | --- | --- | --- |
|  | **Bivariate Model** | | **Multivariate Model** | |
|  | **OR** | **95% CI** | **OR** | **95% CI** |
| **Parental incarceration (ref. = No history of PI)** |  |  |  |  |
| History of PI | 2.069** | [1.23,3.50] | 1.36 | [0.78,2.36] |
| **Age category (ref. = Early Childhood (2 - <6 y))** |  |  |  |  |
| Middle Childhood (6 - <12 y) |  |  | 0.540** | [0.35,0.84] |
| Adolescence (12 - <18 y) |  |  | 0.981 | [0.66,1.46] |
| **Sex (ref. = Male)** |  |  |  |  |
| Female |  |  | 1.092 | [0.77,1.54] |
| **Race/ethnicity (ref. = NH White)** |  |  |  |  |
| Hispanic |  |  | 1.085 | [0.72,1.64] |
| NH Black |  |  | 0.851 | [0.47,1.53] |
| Other (NH Asian, NH AIAN, Other/Multiracial) |  |  | 1.069 | [0.61,1.86] |
| **Number of parents in household (ref. = 2+ parents)** |  |  |  |  |
| No parents in household |  |  | 0.594 | [0.17,2.12] |
| 1 parent |  |  | 1.123 | [0.76,1.65] |
| **Highest level of parent educational attainment (ref. = Post-secondary education)** |  |  |  |  |
| Less than high school |  |  | 0.95 | [0.50,1.81] |
| High school, GED, or equivalent |  |  | 0.957 | [0.59,1.54] |
| **Urbanicity (ref. = Metropolitan)** |  |  |  |  |
| Nonmetropolitan |  |  | 1.3 | [0.89,1.90] |
| **Family income (ref. = Non-Poor [>=200% FPL])** |  |  |  |  |
| Poor (<100% FPL) |  |  | 0.833 | [0.50,1.38] |
| Near-Poor (100% to 199% FPL) |  |  | 0.854 | [0.54,1.34] |
| **Primary source of health insurance (ref. = Private or military)** |  |  |  |  |
| Medicaid, CHIP, or other public |  |  | 2.672*** | [1.71,4.17] |
| Uninsured |  |  | 1.747 | [0.84,3.63] |
| **Washington Group Short Set Composite Disability Indicator (ref. = No)** |  |  |  |  |
| Yes |  |  | 2.895*** | [1.94,4.33] |
| **Intercept** | 0.024*** | [0.02,0.03] | 0.015*** | [0.01,0.02] |
| * p<0.05, ** p<0.01, *** p<0.001 |  |  |  |  |
